# Supplementary material for: Urinary Proteomics of Simulated Firefighting Tasks and Its Relation to Fitness Parameters
Source: Int J Environ Res Public Health. 2021 Oct 11;18(20):10618. doi: 10.3390/ijerph182010618 (PMC8536002; doi:10.3390/ijerph182010618)
Supplement: Supplementary file 1 [file ijerph-18-10618-s001.zip › Supplementary Materials.pdf]

## Supplementary Materials

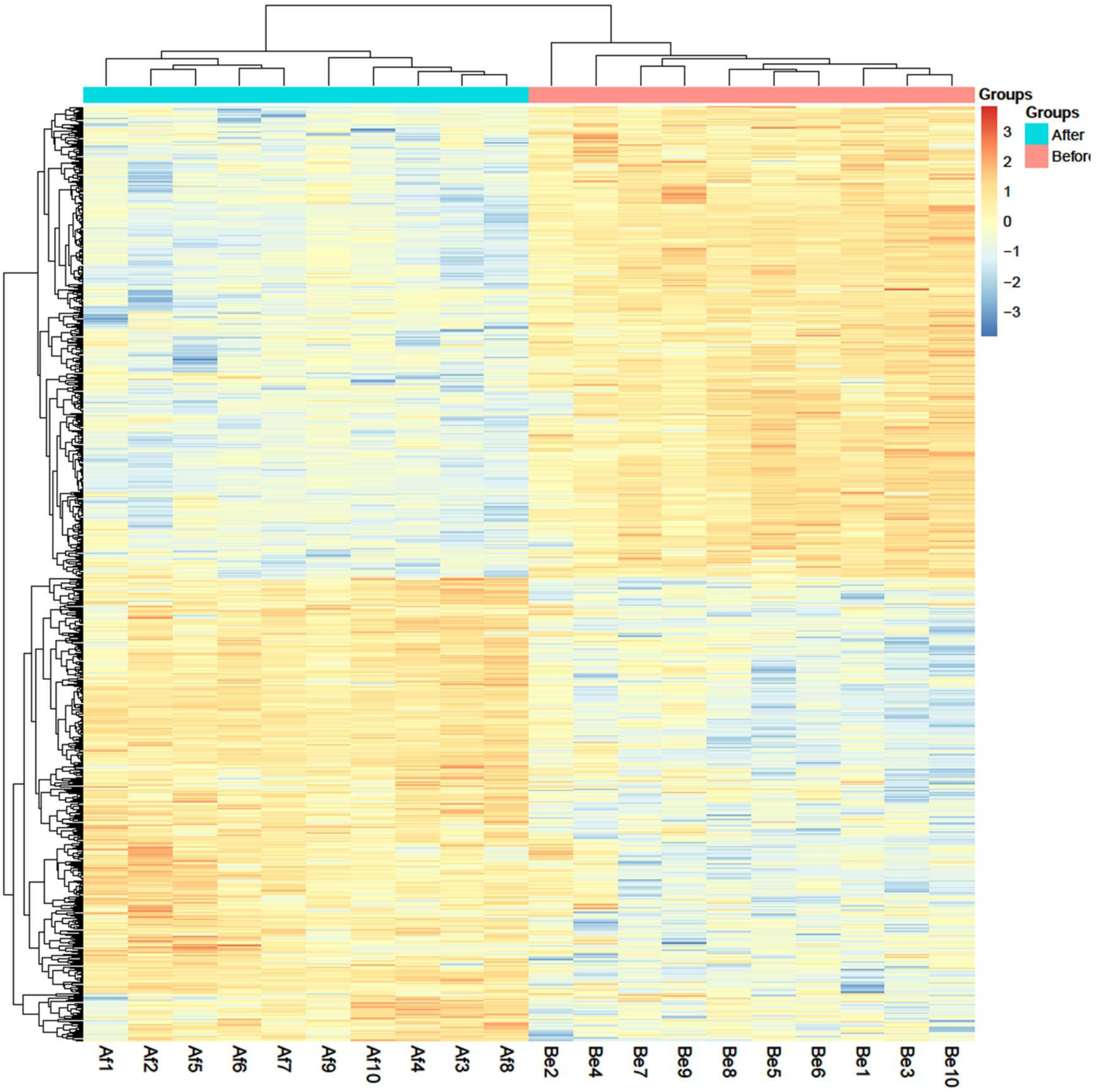


**Figure S1**. Heat map of the proteome data compared before simulated firefighting and after simulated firefighting.

**Table S1.** The KEGG Pathway analysis of the significant differentially expressed proteins.

| ID | the metabolic pathways | Gene Ratio | P value |
|---|---|---|---|
| hsa04610 | Complement and coagulation cascades | 23/283 | <0.001 |
| hsa04142 | Lysosome | 22/283 | <0.001 |
| hsa04514 | Cell adhesion molecules | 21/283 | <0.001 |
| hsa04512 | ECM-receptor interaction | 14/283 | <0.001 |
| hsa03320 | PPAR signaling pathway | 12/283 | 0.004270098 |
| hsa05146 | Amoebiasis | 12/282 | 0.026523 |
| hsa04979 | Cholesterol metabolism | 10/283 | 0.001566071 |
| hsa00531 | Glycosaminoglycan degradation | 5/283 | 0.030694094 |

**Table S2** Top 20 proteins with the most significantly up-regulated or down-regulated.

| ID | Protein | P.adjust value | Fold.Change | Threshold |
|---|---|---|---|---|
| P00915 | Carbonic anhydrase 1 | 0.00000153 | 68.04645 | UP |
| P55808 | Glycoprotein Xg | 0.00000255 | 33.63088 | UP |
| P36955 | Pigment epithelium-derived factor | 0.000000457 | 24.10917 | UP |
| Q9P1F3 | Costars family protein ABRACL | 0.000000267 | 19.40702 | UP |
| Q9UGM5 | Fetuin-B | 0.000000425 | 18.32206 | UP |
| O75368 | SH3 domain-binding glutamic acid-rich-like protein | 0.000000425 | 15.04502 | UP |
| Q08397 | Lysyl oxidase homolog 1 | 0.00000306 | 14.08245 | UP |
| P01008 | Antithrombin-III | 0.0000029 | 13.07405 | UP |
| P61769 | Beta-2-microglobulin | 0.000000375 | 11.41514 | UP |
| P02753 | Retinol-binding protein 4 | 0.000000425 | 8.782076 | UP |
| P01019 | Angiotensinogen | 0.00000211 | 8.079919 | UP |
| Q15848 | Adiponectin | 0.000000267 | 7.67816 | UP |
| P55854;P61956;Q6EEV6 | Small ubiquitin-related modifier 3/2/4 | 0.00000333 | 6.642497 | UP |
| Q13740 | CD166 antigen | 0.000000756 | 6.059273 | UP |
| Q02747 | Guanylin | 0.00000165 | 5.867213 | UP |
| Q14126 | Desmoglein-2 | 0.0000000232 | 4.865698 | UP |
| P01700 | Immunoglobulin lambda variable 1-47 | 0.00000311 | 4.355256 | UP |
| Q92520 | Protein FAM3C | 0.000000425 | 4.253501 | UP |
| Q14766 | Latent-transforming growth factor beta-binding protein 1 | 0.00000159 | 3.682827 | UP |
| P30043 | Flavin reductase (NADPH) | 0.000000457 | 3.573658 | UP |
| P31997 | Carcinoembryonic antigen-related cell adhesion molecule 8 | 0.000041 | 0.477758 | DOWN |
| P04275 | von Willebrand factor | 0.0000325 | 0.463517 | DOWN |
| P53801 | Pituitary tumor-transforming gene 1 protein-interacting protein | 0.0000306 | 0.451365 | DOWN |
| Q9H665 | IGF-like family receptor 1 | 0.0000158 | 0.438283 | DOWN |
| Q99715 | Collagen alpha-1(XII) chain | 0.0000365 | 0.426061 | DOWN |
| Q07954 | Prolow-density lipoprotein receptor-related protein 1 | 0.0000092 | 0.401434 | DOWN |
| Q9NPF0 | CD320 antigen | 0.0000267 | 0.400231 | DOWN |
| P36896 | Activin receptor type-1B | 0.00000311 | 0.397438 | DOWN |
| P24855 | Deoxyribonuclease-1 | 0.0000126 | 0.381064 | DOWN |
| Q68D85 | Natural cytotoxicity triggering receptor 3 ligand 1 | 0.0000312 | 0.380797 | DOWN |
| P24530 | Endothelin receptor type B | 0.0000392 | 0.375717 | DOWN |
| Q6UVK1 | Chondroitin sulfate proteoglycan 4 | 0.0000158 | 0.364728 | DOWN |
| Q7Z7M0 | Multiple epidermal growth factor-like domains protein 8 | 0.0000134 | 0.35714 | DOWN |
| O95998 | Interleukin-18-binding protein | 0.00000919 | 0.320017 | DOWN |
| Q9UKZ9 | Procollagen C-endopeptidase enhancer 2 | 0.00000465 | 0.309874 | DOWN |
| Q6UX71 | Plexin domain-containing protein 2 | 0.00000333 | 0.301267 | DOWN |
| Q71RC9 | Small integral membrane protein 5 | 0.0000165 | 0.253228 | DOWN |
| P09564 | T-cell antigen CD7 | 0.00000311 | 0.245364 | DOWN |
| O75051 | Plexin-A2 | 0.00000631 | 0.235016 | DOWN |
| Q7L0X0 | TLR4 interactor with leucine rich repeats | 0.00000745 | 0.053201 | DOWN |
