# Supplementary figures and images for: Urinary Proteomics of Simulated Firefighting Tasks and Its Relation to Fitness Parameters
Source: Int J Environ Res Public Health. 2021 Oct 11;18(20):10618. doi: 10.3390/ijerph182010618 (PMC8536002; doi:10.3390/ijerph182010618)

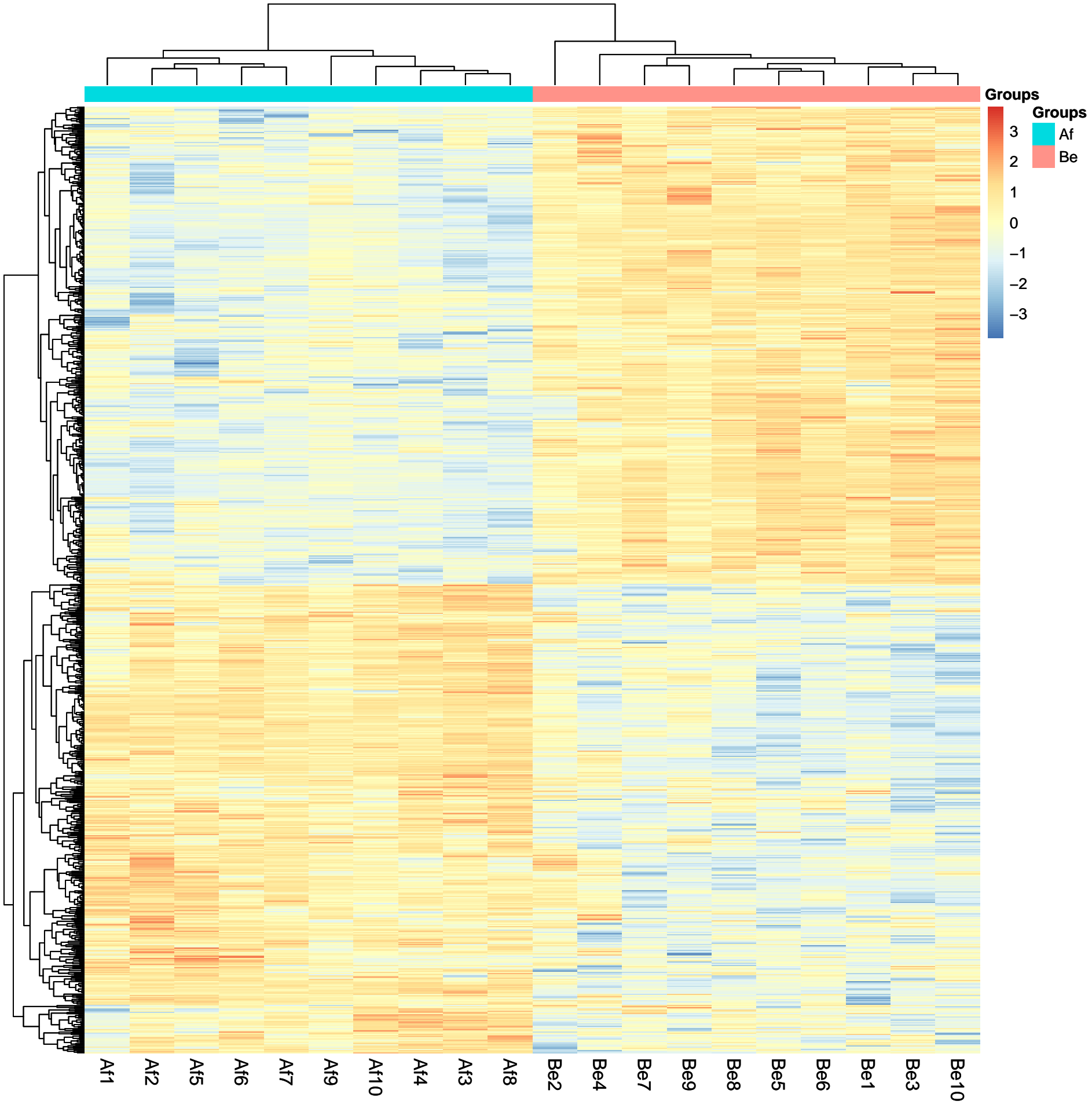

Supplement: Supplementary file 1 [file ijerph-18-10618-s001.zip › Sup 1. HCA_figure.pdf]
